# High rate of house dust mite sensitization in a shrimp allergic southern Ontario population

**DOI:** 10.1186/s13223-017-0177-x

**Published:** 2017-01-19

**Authors:** Lana Rosenfield, Michael William Tsoulis, Kirolos Milio, Meghan Schnittke, Harold Kim

**Affiliations:** 1 Division of Clinical Immunology and Allergy, Department of Medicine Michael D. DeGroote School of Medicine, McMaster University, Hamilton, ON Canada; 2 Department of Obstetrics and Gynecology, McMaster University, Hamilton, ON Canada; 3 Faculty of Science, University of Waterloo, Waterloo, ON Canada; 4 Grandriver Allergy, Kitchener, ON Canada; 5 Schulich School of Medicine & Dentistry, Western University, London, ON Canada

**Keywords:** House dust mite, Shrimp, Tropomyosin, Canadian, Allergen cross-reactivity, Food allergy, Skin test

## Abstract

**Background:**

Shrimp and house dust mite (HDM) allergies are common in Canadians. Often, both of these allergies occur in the same patient. This may be due to homology of tropomyosin or other potentially shared proteins. The aim of our study was to assess the frequency of house dust mite sensitization in a shrimp allergic Canadian population.

**Methods:**

We undertook a retrospective chart review of shrimp allergic patients at an outpatient allergy clinic in Kitchener, Ontario, Canada. Our primary endpoint was to assess for presence of HDM sensitization in this population. Patients were categorized into approximate quartiles. We assessed the severity of the shrimp reactions, correlated shrimp skin test size to HDM skin test size, and measured the proportion of patients with atopic symptoms.

**Results:**

We identified 95 shrimp allergic patients who were tested for house dust mite. 86 (90.5%) of these patients had a positive skin test to HDM. Patients with a shrimp skin test ≥5 mm were 5.31 times (95% CI, 1.55–18.14; p = 0.008) more likely to exhibit a dust mite skin test ≥5 mm than patients with a shrimp skin test <5 mm. The odds of a patient with a shrimp skin test between 10 and 18 mm having a larger HDM skin test were 3.93 times (95% CI 1.03–14.98, p = 0.045) the odds for a patient with a shrimp skin test size between 3 and 4 mm. We did not find a correlation between shrimp skin test size and shrimp reaction symptom grade (p = 0.301).

**Conclusion:**

In our Canadian patients, we found a large majority of shrimp allergic patients to be sensitized to HDM. We found that patients with a large skin test to shrimp were more likely to have a large skin test to HDM compared to those patients with a small skin test to shrimp. We did not find a correlation between shrimp skin test size and shrimp reaction symptom severity. Most of these patients had symptoms of rhinitis and/or asthma that may have been caused by house dust mite allergy.

## Background

Shrimp and house dust mite (HDM) allergies are encountered frequently in Canadian allergy clinics. The prevalence of shellfish allergy in Canada has been found to be 1.6% [1]. There are times when both of these allergies are seen in the same patients, which has been an area of study.

Tropomyosin, a muscle protein, is thought to be responsible for the relationship between HDM and shrimp [2, 3]. It is a protein present in both that shares homology between HDM and shrimp [4]. Tropomyosin in shrimp, referred to as Pen a 1, is the major allergen in shrimp [5, 6] and has been shown to be detected in 41% of patients with shrimp allergy in an Italian population [7]. Der p 10, the tropomyosin found in HDM is present in a minority of patients with HDM allergy [8, 9]. In a study on European patients 9–18% showed IgE reactivity to Der p 10 [10], with some studies showing as low as 4.3% [11] and others up to 25% in adults and 30% in children [12]. Some have postulated these higher levels being in patients with HDM sensitization who live in costal location with more seafood intake [12].

The tropomyosin present in HDM and shrimp has shown cross reactivity [4, 13]. While both shrimp and HDM have tropomyosin, there is variable evidence on showing presence of an allergy to both in patients. There is also evidence which questions tropomyosin as the allergen responsible for cross-reactivity with shellfish, and that other proteins may be a factor [14]. Tropomyosin also has been shown to be responsible for cross reactivity between different crustacea, such as shrimp, crab and lobster, due to homology between the protein in these species [3, 5].

We undertook a retrospective chart review to assess for the presence of HDM sensitization (positive skin test) in shrimp allergic Canadians. The severity of the shrimp reactions was estimated based on patients’ history. We correlated shrimp skin test size to the clinical reaction severity to shrimp. Other objectives included assessing if those shrimp allergic patients who are sensitized to HDM have symptomatic HDM allergy, and for a possible correlation of shrimp skin test size with the presence of HDM allergic symptoms.

## Methods

### Ethics

Ethics was obtained through the Hamilton Integrated Research Ethics Board.

### Patient selection

The charts of patients, with clinical shellfish allergy with positive skin tests to shrimp, who presented to an outpatient allergy clinic in Kitchener, Ontario, Canada were reviewed. The clinical history of the reaction(s), skin prick testing and specific IgE to shrimp and other shellfish was extracted from the available clinical data. Skin test results to aeroallergens including house dust mite was also recorded.

### Skin prick testing

All patients with the question of shrimp allergy underwent skin prick testing testing to crustaceans. Most patients presenting with food allergy to the clinic had testing to the inhalant allergens, including HDM. Reagents for skin prick testing to shrimp were from Omega Laboratories Ltd. Skin prick testing for HDM was done with *D. farinae* and *D. pteronyssinus* at a concentration of 30,000 AU/mL (Hollister-Stier). The largest skin test wheal of *D. farinae* or *D. pteronyssinus* was recorded. The HDM skin test was recorded as negative if both species of HDM tests were negative. A positive result was considered ≥3 mm wheal compared to the negative control test. The results of skin testing were placed into groupings based on wheal diameter.

### Specific IgE

For some of our patients, serum specific IgE testing was done in community labs by ImmunoCap^®^.

### Diagnosing shrimp allergy

Shrimp allergy was diagnosed based on clinical history of an IgE-mediated reaction with positive skin prick testing and/or serum specific IgE levels. The severity of the reaction was categorized based on previously published guides for grading food induced anaphylaxis [15].

### Statistical analysis

All statistical analyses were carried out with SPSS software (Version 23; IBM, Chicago, Ill). The χ^2^ test for Independence or Fisher’s exact test for Independence, where appropriate, was used to analyze the association between 2 categorical variables. Independent-sample Student t test was used to compare the mean score of continuous data between categorical groups, namely patients allergic to shrimp with sensitization to dust mite versus patients allergic to shrimp without sensitization to dust mite. Binary logistic regression was used to estimate the influence of shrimp allergy on dust mite allergy. The dependent variable was skin test to dust mite (≥5 mm = 1 and <5 mm = 0) and explanatory variable was skin test to shrimp (≥5 mm = 1 and <5 mm = 0). To better ascertain the relationship between dust mite and shrimp sensitization, an ordinal logistic regression was used to estimate the influence of shrimp sensitization severity on dust mite sensitization severity. Both shrimp and dust mite skin test values were apportioned into quartiles: Shrimp, 25th, 50th, and 75th at 5, 7, and 10 mm, respectively; Dust mite, 25th, 50th, and 75th at 3, 5, and 7 mm, respectively. Quartered shrimp skin test values were used as the explanatory variable and quartered dust mite skin test values were used as the dependent variable. Odds ratios (ORs) were reported with corresponding 95% confidence intervals (95% CIs). All tests were 2-sided and p values <0.05 were considered statistically significant.

## Results

### Demographics and diagnosis of shrimp allergy

We identified 95 patients with shrimp allergy that were also tested to HDM. The demographic data of these patients along with presence of other crustacean and inhalant allergies and symptoms of atopy can be found in Table 1. There was no significant difference between the size of skin test to shrimp and average of grade of symptoms to shrimp ingestion between those with and without dust mite sensitization (Table 1).

**Table 1:** Demographic characteristics

	Shrimp allergy & + HDM	Shrimp allergy & − HDM	p value
n	86	9	
Age	29.40 ± 2.08	17.78 ± 4.82	0.083
Sex			
Male (%)	55 (59)	5 (56)	0.935
Female (%)	39 (41)	4 (44)	
Shrimp skin test (mm)	7.07 ± 0.29	7.22 ± 0.70	0.871
Shrimp symptoms (grade 1–5)	2.36 ± 0.13	2.56 ± 0.44	0.685
Lobster allergy (%)	50 (82)^a^	7 (100)^b^	0.588
Crab allergy (%)	37 (62)^a^	3 (43)^b^	0.427
Other food allergy (%)	27 (31)	3 (33)	0.905
Ragweed allergy (%)	49 (57)	7 (78)	0.300
Dog allergy (%)	36 (42)	4 (44)	0.881
Cat allergy (%)	57 (66)	7 (78)	0.714
Alternaria allergy (%)	30 (35)	2 (22)	0.713
Grass allergy (%)	56 (65)	8 (89)	0.263
Asthma (%)	40 (47)	5 (56)	0.731
Allergic rhinitis (%)	55 (64)	6 (67)	0.872
Atopic dermatitis (%)	14 (16)	1 (11)	0.686

Demographic data for shrimp allergic patients with positive (+) or negative (−) skin test to house dust mite (HDM). Also contains presence of other crustacean allergy, inhalant allergy, other food allergy and symptoms of atopy (asthma, allergic rhinitis and atopic dermatitis)

Data are presented as mean ± SEM where appropriate

Percent of n in parentheses

^a^n does not equal 86 (n = 61)

^b^n does not equal 9 (n = 7)

### HDM sensitization in Shrimp allergic patients

**Fig. 1:**
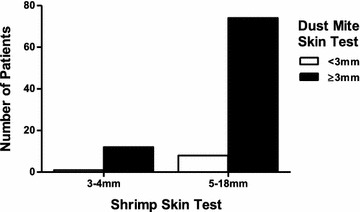
Comparison of skin test size to house dust mite by shrimp skin test size

**Fig. 2:**
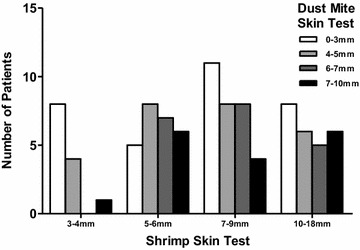
Approximate quartiles of patient skin test to shrimp compared to number of patient in HDM quartiles

Of the 95 patients with shrimp allergy, 90.5% had positive skin testing to HDM. The correlation between HDM skin test size and shrimp skin test size was not significant (r = −0.109, p = 0.293; pearson correlation). The relationship between HDM skin test (<3 or ≥3 mm) and shrimp skin test (≥5 mm and <5 mm) was not significant (*p* = 1.000). We found the odds ratio of having a HDM test greater than 3 mm, with shrimp skin test size ≥5 mm versus <5 mm, was 0.771 (95% CI 0.088–6.73, p = 0.814). Figure 1 shows the number of patients with a skin test size to shrimp ≥5 mm who have positive (≥3 mm) skin test to dust mite. While there is a majority of patients in this category, too few patients in our population had <3 mm dust mite skin tests for a meaningful analysis. Therefore, the data was further categorized into those patients with a HDM skin test size ≥5 mm and those <5 mm to determine if there was an association between shrimp skin test size (≥5 mm and <5 mm). The relationship between HDM skin test (≥5 mm or <5 mm) and shrimp skin test (≥5 mm or <5 mm) was significant, χ^2^ (1) = 8.12, *p* = 0.004. Patients with a shrimp skin test of ≥5 mm were more likely than patients with a shrimp skin test of <5 mm to have a HDM skin test of ≥5 mm. We found that patients with a shrimp skin test ≥5 mm were 5.31 times more likely to exhibit a HDM skin test ≥5 mm (95% CI 1.55–18.14, p = 0.008) than patients with a shrimp skin test <5 mm. Figure 2 shows the majority of positive HDM skin tests in patients with shrimp skin tests >5 mm. Interestingly, there are patients negative to HDM in all quartiles and there does not appear to be a greater number of patients with larger skin test to HDM in the larger shrimp skin test group.

The odds of a patient with a shrimp skin test size between 5 and 6 mm having a larger dust mite skin test were 5.49 times (95% CI 1.43–21.02, p = 0.013) the odds for a patient with a shrimp skin test size between 3 and 4 mm. The odds of a patient with a shrimp skin test size between 7 and 9 mm of having a larger HDM skin test were 2.68 times compared to a patient with a shrimp skin test size between 3 and 4 mm; however, this did not reach statistical significance (95% CI 0.74–9.73, p = 0.134). The odds of a patient with a shrimp skin test between 10 and 18 mm having a larger HDM skin test were 3.93 times (95% CI 1.03–14.98, p = 0.045) the odds for a patient with a shrimp skin test size of 3–4 mm.

### Skin test size to shrimp compared to the symptom severity of shrimp reactions

This data also allowed us to assess the correlation between size of shrimp skin test and severity of shrimp reaction. Table 2 shows the symptom grade [15] to shrimp ingestion by skin test size. There are 5 extra patients included in this table and analysis. These patients did not have HDM testing done so were not included in our primary analysis, but did have symptoms with shrimp and skin test to shrimp along with recording of atopy symptoms. The correlation between shrimp skin test size and shrimp symptom grade was not significant (r = −0.102, p = 0.301; Pearson correlation). The odds ratio of having a higher shrimp symptom grade, with a shrimp skin test size between 5 and 6 mm versus 3 and 4 mm, was 1.79 (95% CI 0.54–5.93, p = 0.337) and not significant. The odds ratio of having a high shrimp symptom grade, with a shrimp skin test size between 7 and 9 mm versus 3–4 mm, was 1.09 (95% CI 0.34–3.51, p = 0.337) and not significant.

**Table 2:** Symptoms of shrimp ingestion, dust mite sensitization and atopic symptoms based on approximate quartiles of shrimp skin test diameter

	Shrimp skin test				p value
	3–4 mm	5–6 mm	7–9 mm	10–18 mm	
n = 100	13	27	31	29	
Shrimp symptoms severity grade 1–5 (%)					
1	3 (23)	8 (30)	12 (39)	12 (41)	
2	7 (54)	2 (7)	7 (23)	4 (14)	
3	1 (8)	9 (33)	3 (10)	7 (24)	
4	2 (15)	8 (30)	9 (29)	6 (21)	0.077
5	0	0	0	0	
Presence of asthma (%)	6 (46)	13 (48)	15 (48)	13 (45)	0.992
Presence of allergic rhinitis (%)	8 (62)	17 (63)	18 (58)	19 (66)	0.947
Presence of dermatitis (%)	2 (15)	6 (22)	3 (10)	5 (17)	0.608

Approximate quartiles of shrimp skin testing showing presence of atopy, skin test positivity to dust mite and grade of shrimp ingestion symptoms

The odds ratio of having a higher shrimp symptom grade, with a shrimp skin test size between 10 and 18 mm versus 3–4 mm, was 0.997 (95% CI 0.31–3.26, p = 0.996) and not significant.

### Presence of atopic symptoms in shrimp allergic patients

Many of our patients with shrimp allergy had atopic symptoms. In Table 1 it can be seen that there are symptoms of asthma, allergic rhinitis and atopic dermatitis in both HDM and non-HDM sensitized shrimp allergic patients. There was no significant difference between the presence of these symptoms in those HDM versus non-HDM sensitized. When breaking down all shrimp allergic patients into approximate quartiles (Table 2), there is no obvious relationship between size of allergy skin test to shrimp and the presence of asthma, allergic rhinitis and dermatitis.

## Discussion

Shrimp allergy is seen in more than 1% of the Canadian population [1]. Our results demonstrate that almost all of our patients with shrimp allergy were also sensitized to house dust mite. This is similar to small studies on an Asian population which have found a majority of patients with shrimp allergy have positive skin tests to HDM. One demonstrated that patients who identify as shrimp allergic, both those that react on oral food challenge and those who can tolerate shrimp, are almost all positive on skin testing to HDM [16]. Another showed that 72% of shellfish sensitized individuals have skin test positivity to HDM [17].

Alternatively when looking at shrimp sensitization in those with HDM positivity, the minority show positivity to shrimp [16]. This may be explained by the role of tropomyosin in the shrimp and HDM allergies. Tropomyosin is the major allergen in shrimp [5, 6] and therefore, sensitization to the protein would be likely found in a majority of shrimp allergic individuals. These patients would have a greater potential to cross react with Der p 10 of HDM. This would explain why we, along with other groups, have found a high frequency of HDM sensitization in shrimp allergic patients. When looking at patients with HDM allergy for shrimp sensitization, Der p 10 is not the major allergen in HDM allergy. Therefore, it may be expected that the number of patients who are HDM allergic with shrimp sensitization would be lower, as a minority of patients with HDM allergy are sensitized to the tropomyosin. A previous study found that HDM allergic patients were more likely to be Der p 10 negative with a HDM allergy alone compared to combined HDM and shrimp allergy, which did have higher levels of Der p 10 IgE [12].

We found there to be an increased odds of having a sizeable HDM skin test wheal size (≥5 mm) if patients also had a shrimp skin test wheal size (≥5 mm) compared to patients with a smaller shrimp skin test wheal size (<5 mm). Interestingly, a previous study showed there was a non-significant smaller wheal size to *D. pteronyssinus* in patients with seafood allergy than those without seafood allergy [18]. While we did not compare the wheal size for HDM in shrimp allergic patients to those not shrimp allergic, a larger HDM skin test (≥5 mm) was more likely if the patient had a large shrimp skin test (≥5 mm).

Based on the protein homology theory, HDM allergic individuals would be expected to be sensitized to the tropomyosin component of HDM for cross reactivity to occur. We did not have the ability to look into the specific components (Der p 10 and Pen a 1) of our patients’ HDM and shrimp allergy to find sensitization to tropomyosin. Interestingly, there are studies of patients who do not have elevated levels of specific IgE to tropomyosin that are positive to dust mite who develop symptoms after consuming shellfish [18]. This demonstrates that there may be alternative allergenic proteins involved with the cross-reactivity between these two allergens, and some alternative allergens have been identified [14].

An alternative explanation for the high frequency of HDM sensitization in our patients may be high HDM exposure and coincidental HDM sensitization. Increased HDM exposure may result in increased sensitization to HDM [19]. There is variable evidence for different factors such as humidity or damp house environment as a cause of increased household HDM. A Canadian study assessed this idea, and while there was higher amounts of HDM in Vancouver (which is more humid) compared to Winnipeg, the relative indoor humidity level was not a significant factor for the higher levels of HDM in Vancouver [20]. Our patients, from Southern Ontario, also live in an area of increased humidity. But based on the aforementioned evidence, increased outdoor humidity may not lead to increased HDM sensitization. To further characterize this, we would need to compare a control group (perhaps patients with food allergies other than shellfish) to compare the levels of HDM sensitization in our area.

Our data also allowed us to look for a correlation between size of skin test and severity of reaction to shrimp. There was not a significant relationship, which was confirmed when looking at the shrimp skin test quartiles by ordinal logistic regression. These results are similar to previous studies which found no association between skin test size and clinical reaction severity in food allergy (shrimp was not included in this study) [21].

One limitation of our study was that it was completed in only one clinic in Southern Ontario with a limited sample size. The findings may differ in a multicenter study. The location of this study may be a factor as high humidity in Southern Ontario was likely a factor in the high proportion of HDM sensitization. Also, we did not perform oral challenges to shrimp in our patients to confirm shrimp allergy. It is likely that some patients would have passed an oral challenge so they would have been excluded from the analysis. And as noted previously, we did not perform serum shrimp specific IgE or HDM specific IgE levels on all of our patients. Further objective data would have been interesting to analyze.

Future research should address a number of questions that this study has generated. We would like to see if our patients would have a resolution of shrimp allergy if they were treated with HDM immunotherapy. There have been previous cases showing that food allergy symptoms to shrimp have resolved after HDM immunotherapy. There is conflicting evidence for development of shrimp allergy with dust mite immunotherapy. One study, where subcutaneous immunotherapy was given to HDM allergic patients who were not sensitized to shrimp, showed that patients did not develop positive skin test or symptoms with shellfish consumption [22]. Another study, assessing specific IgE to Pen a 1 before and after sublingual immunotherapy to HDM, did not find any patients who formed antibodies to shrimp tropomyosin [23]. Alternatively, there is a study that showed an increase in shrimp IgE in some patients after receiving dust mite immunotherapy [24]. Other studies could assess whether different areas of the country have different levels of HDM sensitization in shrimp allergic patients. One may postulate that the coastal areas or more humid areas would have a higher proportion of these patients and dry areas fewer. Also, as previously mentioned, it would be fascinating to look at the HDM sensitization in all food allergic patients and compare this to shrimp allergic patients.

## Conclusion

In conclusion, we found a large majority of southern Ontario shrimp allergic patients to be sensitized to HDM. There was a correlation with having a larger skin test to house dust mite in patients with a larger skin test to shrimp. We were not able to find any relationship between skin test size to shrimp and the severity of shrimp reaction, which emphasizes the poor ability of skin test to predict reaction severity with food allergy.

## Abbreviation

HDM: house dust mite
